# Supplementary material for: Endothelial PTBP1 Deletion in Transplanted Cardiac Tissue Limits Cardiac Allograft Vasculopathy
Source: bioRxiv. 2026 Feb 19:2026.02.18.706637. Preprint. [Version 1] doi: 10.64898/2026.02.18.706637 (PMC12934740; doi:10.64898/2026.02.18.706637)
Supplement: Supplement 1 [file NIHPP2026.02.18.706637v1-supplement-1.pdf]

bioRxiv preprint doi: https://doi.org/10.64898/2026.02.18.706637; this version posted February 19, 2026. The copyright holder for this preprint (which was not certified by peer review) is the author/funder, who has granted bioRxiv a license to display the preprint in perpetuity. It is made available under aCC-BY 4.0 International license.

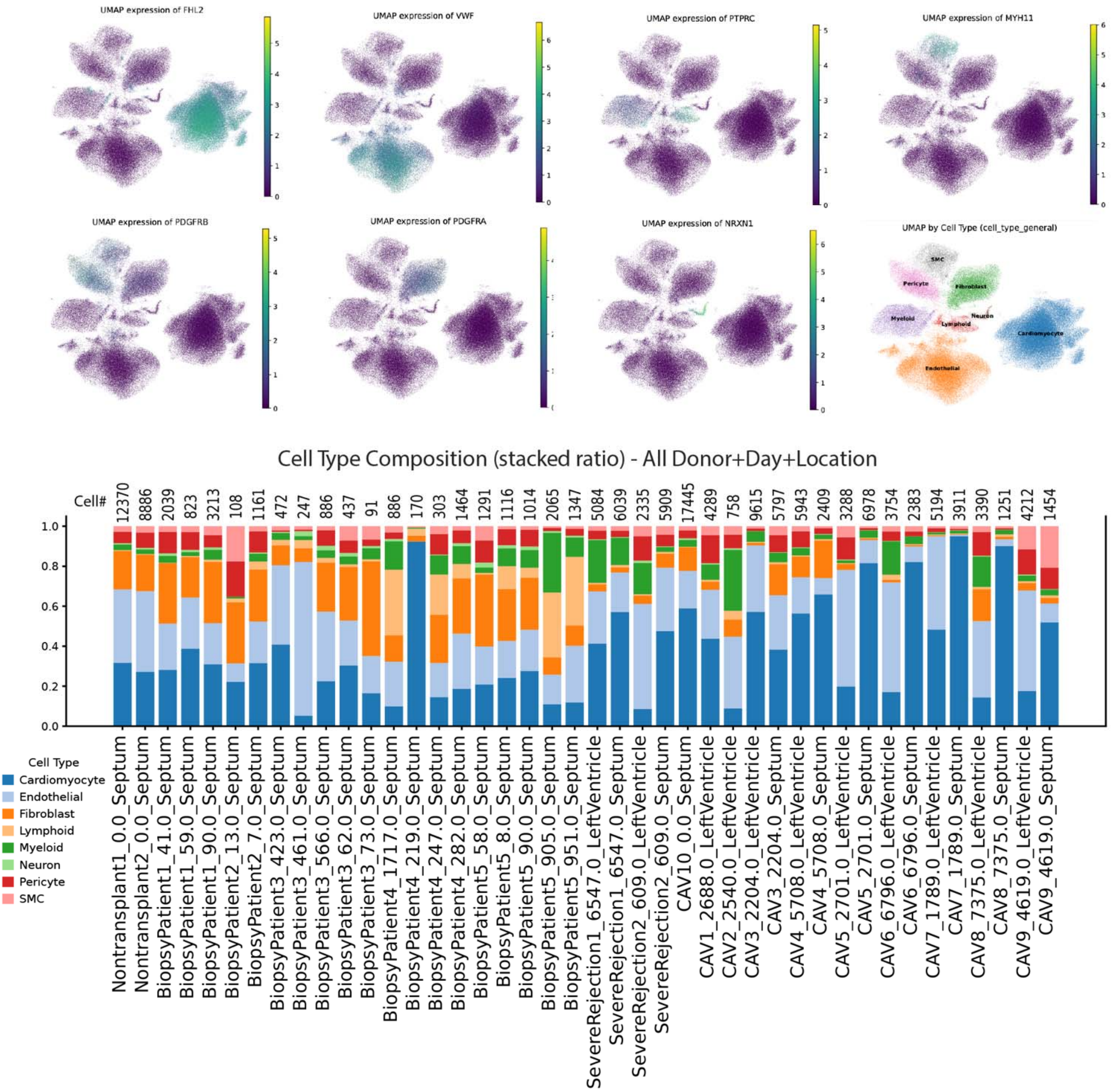

SI Figure 1. Single cell analysis showing basic characterization. UMAP clusters showing the expression of key cluster marker genes, across all samples. Barplots below show individual samples, labeled by sample ID, day post explant, and location. Total cell number in each sample is shown at the top.

bioRxiv preprint doi: https://doi.org/10.64898/2026.02.18.706637; this version posted February 19, 2026. The copyright holder for this preprint (which was not certified by peer review) is the author/funder, who has granted bioRxiv a license to display the preprint in perpetuity. It is made available under aCC-BY 4.0 International license.

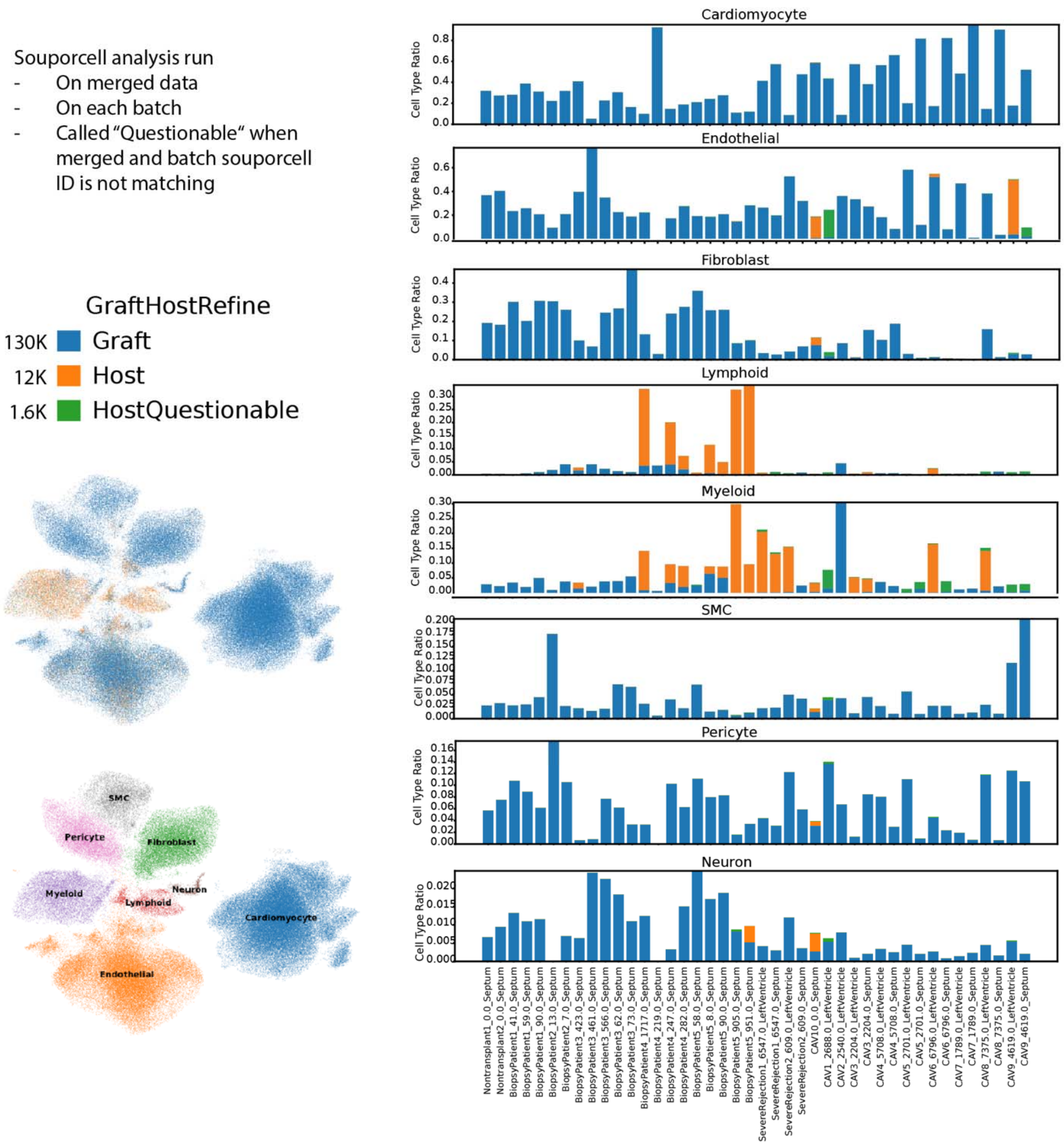

SI Figure 2. Analysis of graft versus host status by SouporCell de novo SNP analysis. Analysis of graft versus host status in single cell data by Souporcell. Analysis was run on each batch individually, and to provide more power and to extend one ID between batches, on all batches joined together. Questionable calls are those where call by batch and across all batches did not align. Bar plots show fraction of each within each population. Each sample is labeled by donor, day, and location.

bioRxiv preprint doi: https://doi.org/10.64898/2026.02.18.706637; this version posted February 19, 2026. The copyright holder for this preprint (which was not certified by peer review) is the author/funder, who has granted bioRxiv a license to display the preprint in perpetuity. It is made available under aCC-BY 4.0 International license.

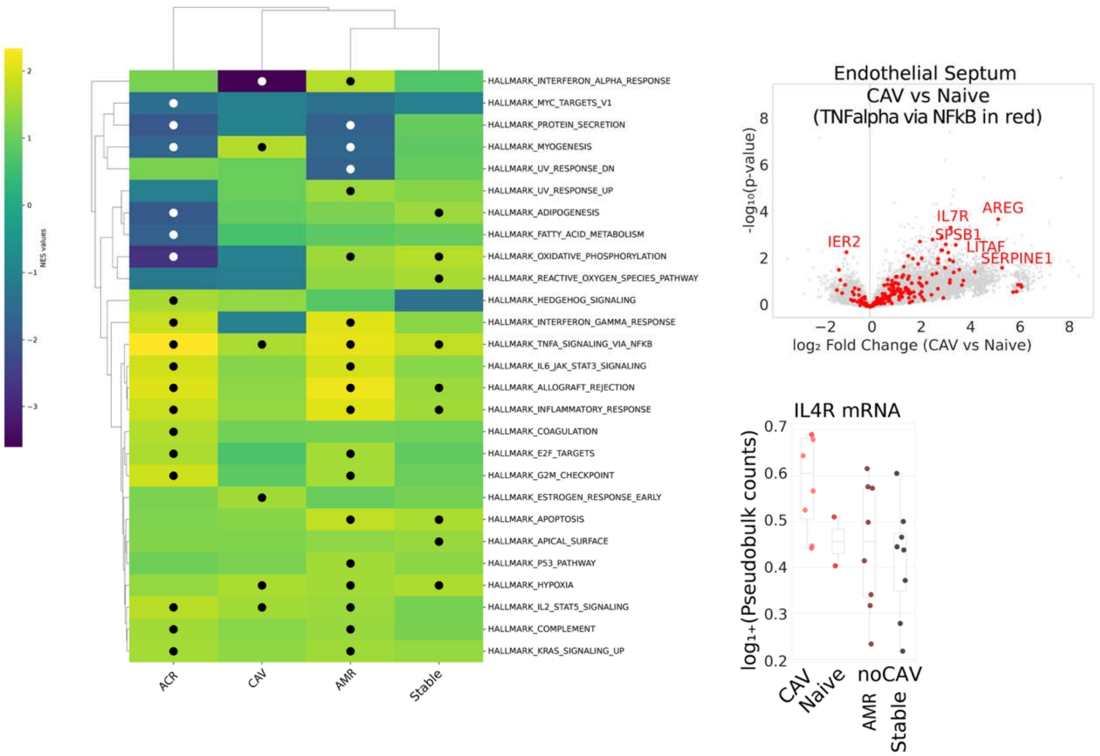

SI Figure 3. Gene set enrichment in endothelium, comparing samples to naïve heart endothelium. DESeq was used to examine differences between stable biopsies or unaffected heart tissues and CAV tissues for each cell population. Gene set enrichment was performed, using DESeq2 stat, to determine increased and decreased expression of gene sets. Circles = padj < 0.05. Volcano plot showing altered gene expression in the endothelial cell clusters (pseudobulk) in septal samples from CAV-positive explant versus naïve, with overlay of genes associated with the specific TNF-alpha mSigDB Hallmark pathway. Targeted analysis of IL4R showing differential expression, in quartile normalized read counts in each endothelial sample. CAV-negative biopsies were further separated into samples with mild rejection (Rejecting) or without signs of antibody or cellular rejection (Stable).

bioRxiv preprint doi: https://doi.org/10.64898/2026.02.18.706637; this version posted February 19, 2026. The copyright holder for this preprint (which was not certified by peer review) is the author/funder, who has granted bioRxiv a license to display the preprint in perpetuity. It is made available under aCC-BY 4.0 International license.

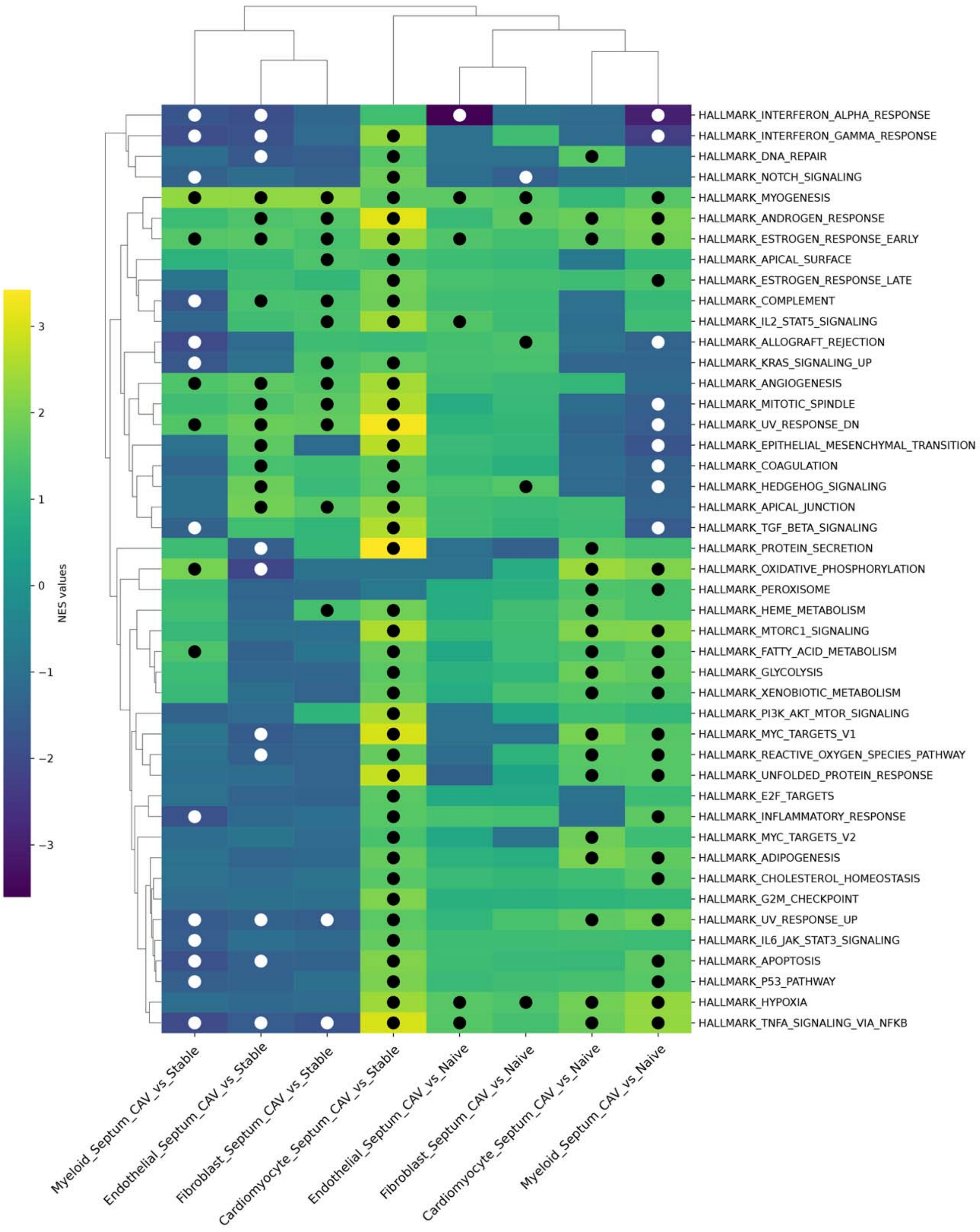

SI Figure 4. Gene set enrichment following pseudobulk analysis of all cells by major cell type. DESeq was used to examine differences between stable biopsies or unaffected heart tissues and CAV tissues for each cell population. Gene set enrichment was performed, using DESeq2 stat, to determine increased and decreased expression of gene sets. Circles = padj < 0.05.

bioRxiv preprint doi: https://doi.org/10.64898/2026.02.18.706637; this version posted February 19, 2026. The copyright holder for this preprint (which was not certified by peer review) is the author/funder, who has granted bioRxiv a license to display the preprint in perpetuity. It is made available under aCC-BY 4.0 International license.

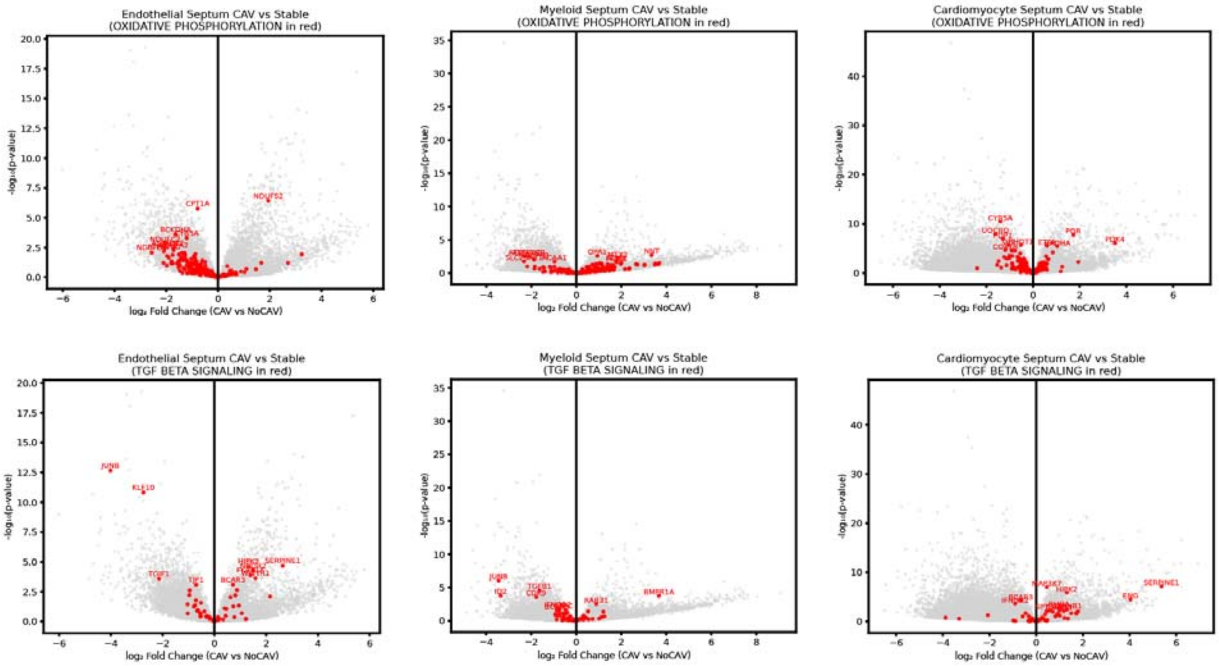

SI Figure 5. Volcano plots showing alterations in indicated gene sets. DEseq2 was used to determine altered expression patterns between stable biopsies the CAV tissues for each cell population. Red indicate the location of genes in the indicated pathways on these plots.

bioRxiv preprint doi: https://doi.org/10.64898/2026.02.18.706637; this version posted February 19, 2026. The copyright holder for this preprint (which was not certified by peer review) is the author/funder, who has granted bioRxiv a license to display the preprint in perpetuity. It is made available under aCC-BY 4.0 International license.

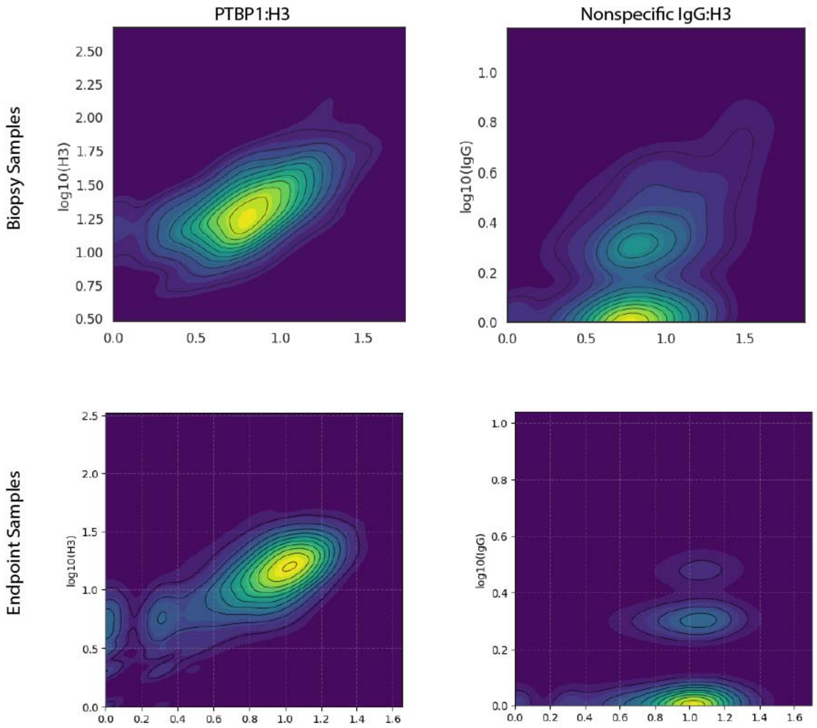

SI Figure 6. inCITE-seq protein counts. Raw protein counts per nuclei are show for each antibody. Percentages indicate the fraction of cells with 0 counts for the antibody. X-Y plots show the correlation between PTBP1 and histone H3 or IgG and histone H3.

bioRxiv preprint doi: https://doi.org/10.64898/2026.02.18.706637; this version posted February 19, 2026. The copyright holder for this preprint (which was not certified by peer review) is the author/funder, who has granted bioRxiv a license to display the preprint in perpetuity. It is made available under aCC-BY 4.0 International license.

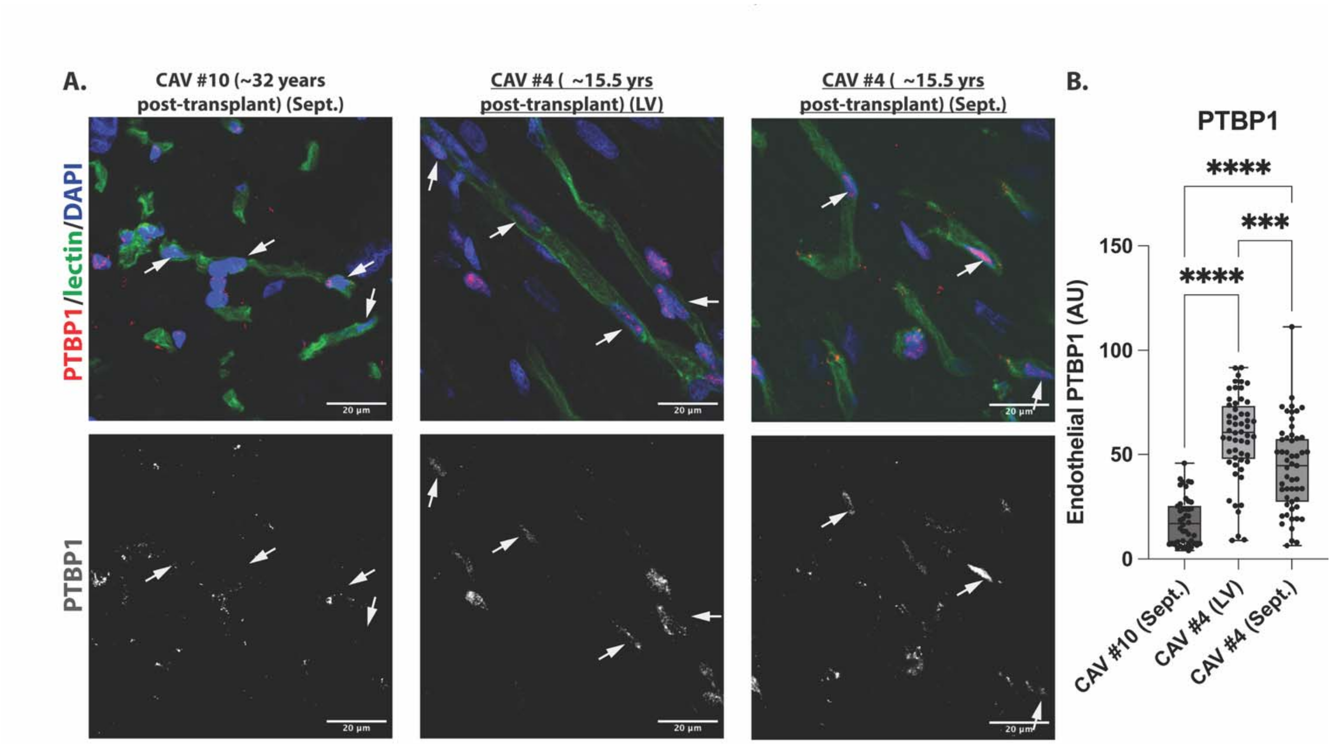

SI Figure 7. PTBP1 staining of long-lasting heart graft. (A) Immunofluorescence staining, showing PTBP1 levels in DAPI+ nuclei associated with UAE-1 lectin-stained vasculature (top panel). CAV tissues are from long-lasting CAV#10 sample (32 yrs post-transplant) or CAV#4 sample (15.5 years post-transplant) and either left ventricular (LV) or septal (Sept.). Bottom panel: grayscale PTBP1 staining alone (B) Quantification of endothelial PTBP1 levels across samples. Dots represent single cells from three random high-powered fields per sample. Statistical significance was assessed by one-way ANOVA with Tukey's multiple comparisons test.

bioRxiv preprint doi: https://doi.org/10.64898/2026.02.18.706637; this version posted February 19, 2026. The copyright holder for this preprint (which was not certified by peer review) is the author/funder, who has granted bioRxiv a license to display the preprint in perpetuity. It is made available under aCC-BY 4.0 International license.

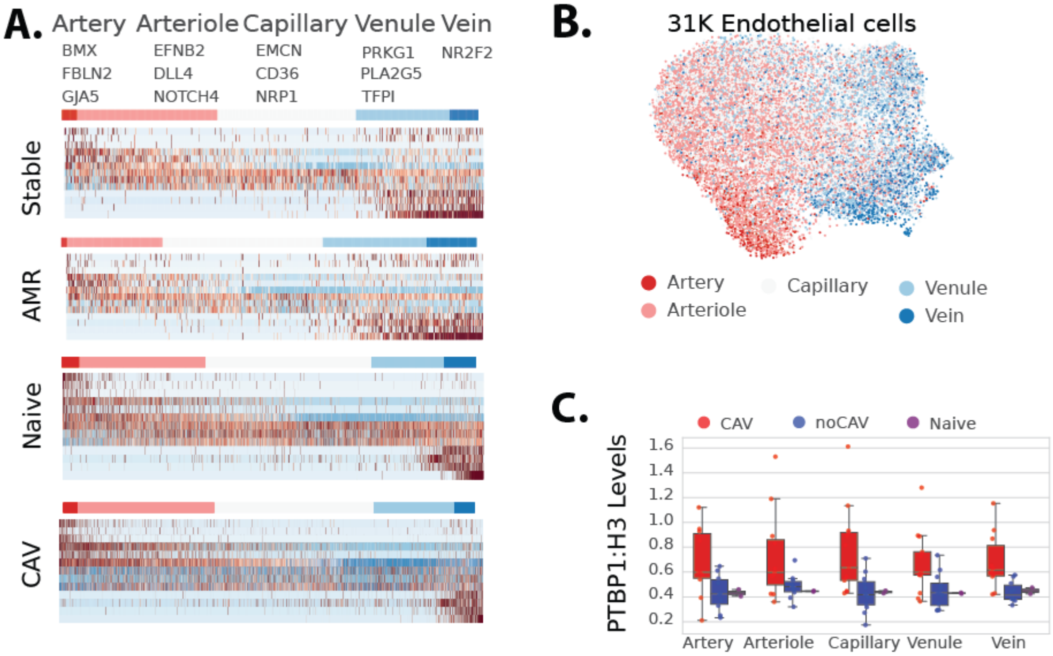

SI Figure 8. inCITE-seq PTBP1 counts across the AV-hierarchy. (A) Heat map aligning endothelial cells along arteriovenous heriarchy, based on scoring of arterial and venous genes (above). (B) Categorization of cells in capillary cluster. (C) PTBP1 counts, normalized to H3, from inCITE-seq data in cells within each region of the arteriovenous hierarchy.

bioRxiv preprint doi: https://doi.org/10.64898/2026.02.18.706637; this version posted February 19, 2026. The copyright holder for this preprint (which was not certified by peer review) is the author/funder, who has granted bioRxiv a license to display the preprint in perpetuity. It is made available under aCC-BY 4.0 International license.

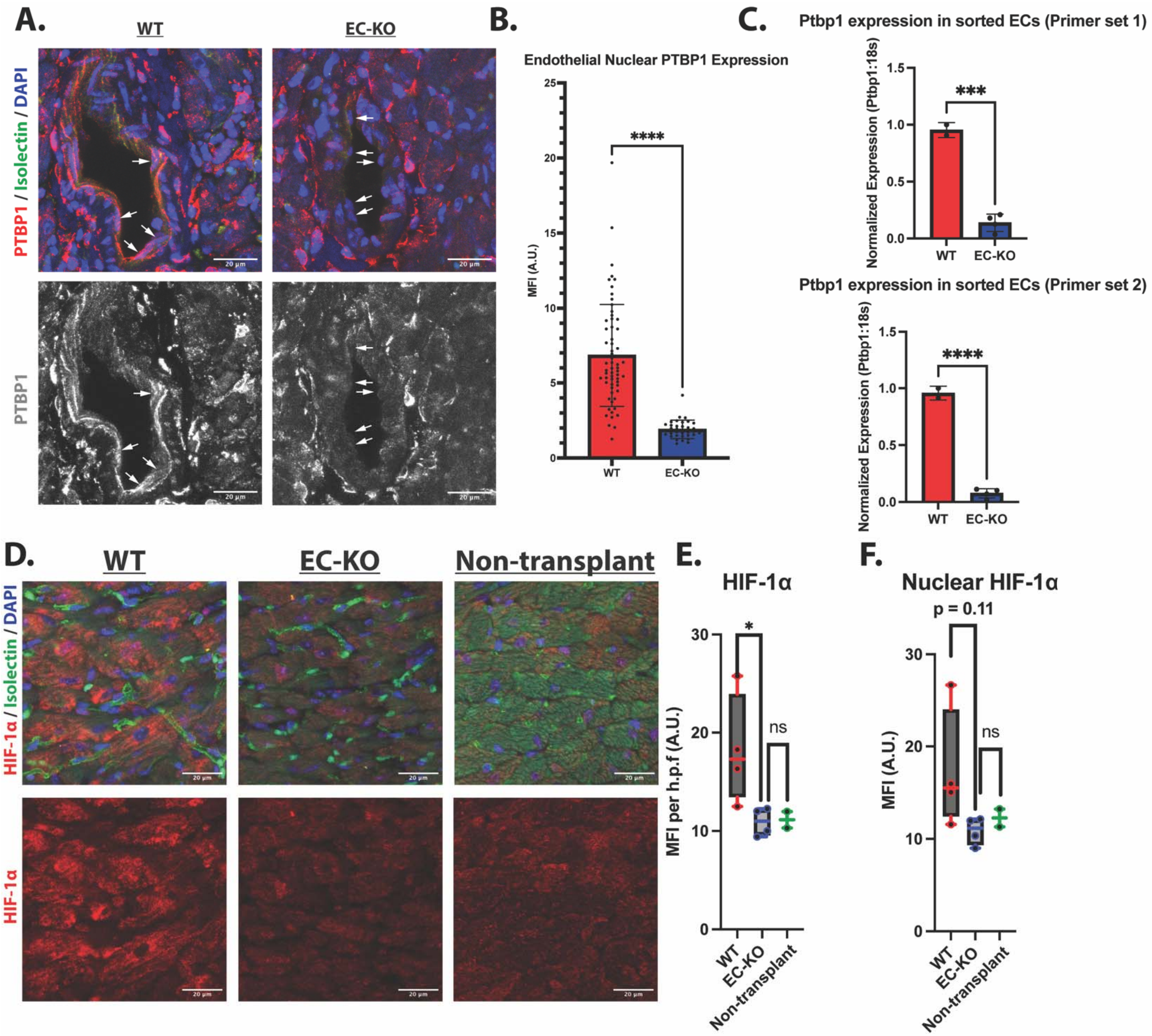

SI Figure 9. PTBP1 and HIF-1α staining of heart grafts. (A) Representative images of WT and EC-KO transplanted hearts stained for PTBP1 (red), isolectin B4 (green), and DAPI (blue). Bottom panels show grayscale single-channel PTBP1 images. (B) Quantification of endothelial nuclear PTBP1 expression in WT (n = 4) and EC-KO (n = 4) grafts. Each dot represents an individual endothelial cell quantified from three images per sample (>20 endothelial cells per sample). Data are shown as mean ± SD; significance was assessed using Mann-Whitney test (****p<0.0001). (C) Quantitative PCR analysis of Ptbp1 expression in CD31+ICAM2+ sorted endothelial cells isolated from WT (n = 2) and EC-KO (n = 4) grafts post-transplant, using two independent primer sets. Statistical significance was assessed by Student's t test (D, E,F) Representative images (D) and quantification of overall mean fluorescence intensity (E) and nuclear mean fluorescence intensity (F) of HIF-1α in WT (n = 4) and EC-KO (n = 4) grafts and in non-transplanted control hearts (n = 2). Data are presented as median, min, and max. Statistical significance was determined using Mann-Whitney test (*p < 0.05).

bioRxiv preprint doi: https://doi.org/10.64898/2026.02.18.706637; this version posted February 19, 2026. The copyright holder for this preprint (which was not certified by peer review) is the author/funder, who has granted bioRxiv a license to display the preprint in perpetuity. It is made available under aCC-BY 4.0 International license.

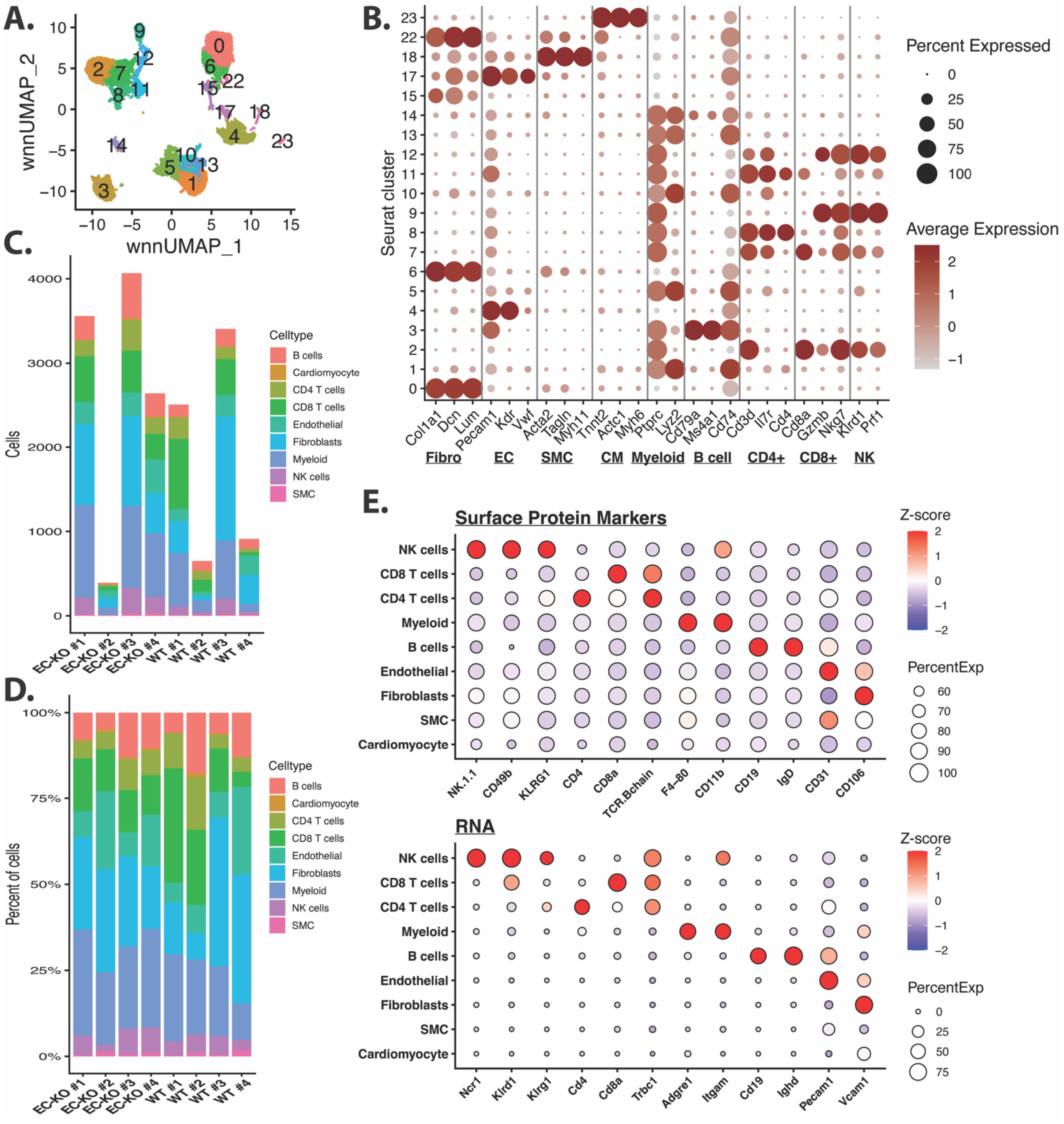

SI Figure 10. Cardiac cell composition of WT and EC-KO grafts by CITE-seq. (A) Weighted nearest neighbor (WNN) RNA–protein integrated UMAP depicting cardiac cell clusters identified by CITE-seq. (B) Dot plot showing canonical RNA markers used to annotate cardiac cell clusters. (C) Number of cells per sample, stratified by cell type. (D) Proportion of each cardiac cell type within individual samples. (E) Dot plot displaying RNA and surface protein expression of canonical markers across major cardiac cell types.

bioRxiv preprint doi: https://doi.org/10.64898/2026.02.18.706637; this version posted February 19, 2026. The copyright holder for this preprint (which was not certified by peer review) is the author/funder, who has granted bioRxiv a license to display the preprint in perpetuity. It is made available under aCC-BY 4.0 International license.

SI

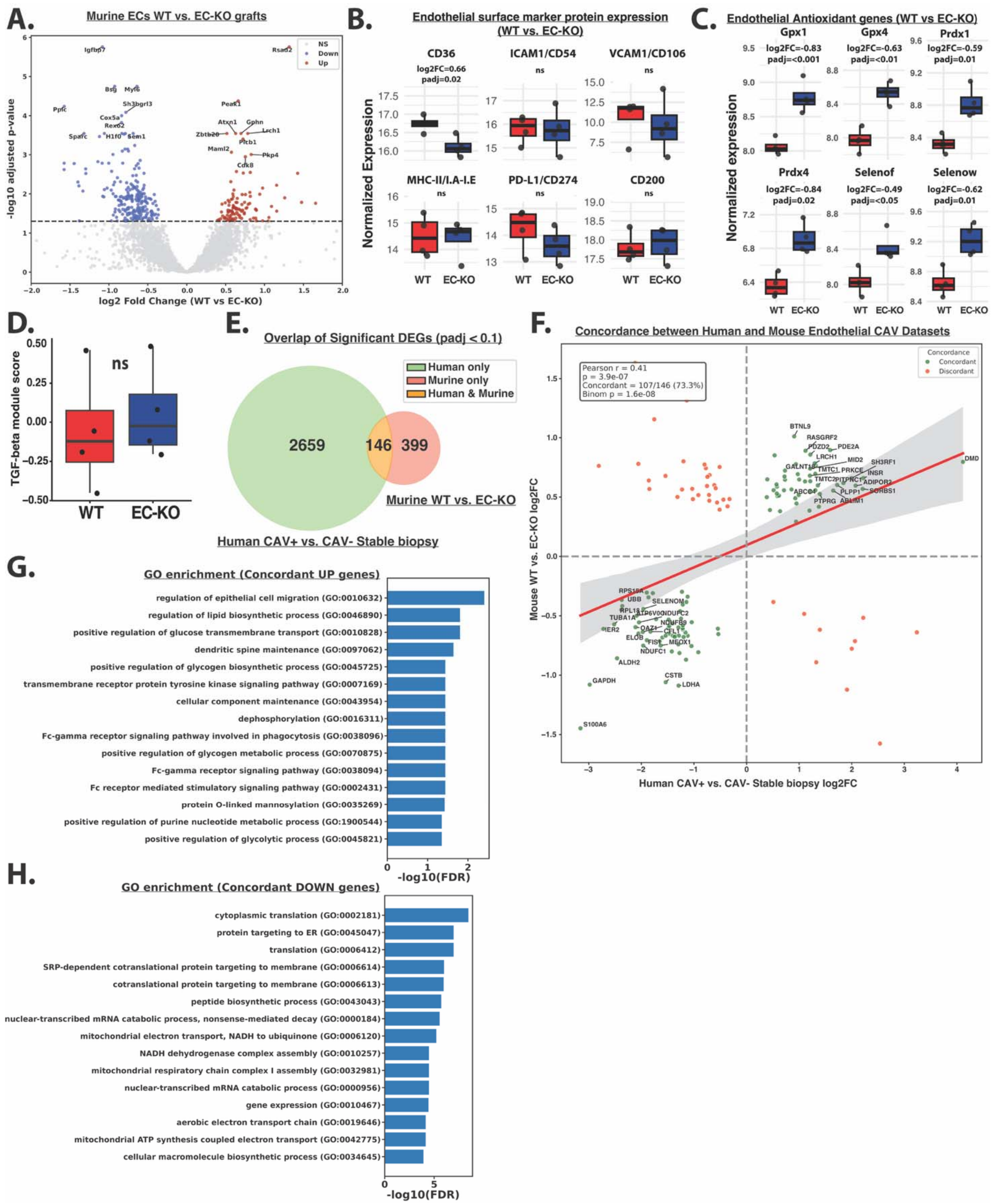

Figure 11. Endothelial cell transcriptional analysis. (A) Volcano plot of pseudobulk differentially expressed genes (DEGs) comparing WT and EC-KO endothelial cells. Significantly upregulated genes are shown in red and downregulated in blue. (B) Pseudobulk expression of select surface protein markers in endothelial cells from WT and EC-KO grafts. Bar plots represent variance-stabilizing transformation (VST)-normalized expression, log2FC and adjusted p-values were calculated using DESeq2. (C) Pseudobulk expression of select antioxidant genes in endothelial cells from WT and EC-KO grafts. Bar plots represent variance-stabilizing transformation (VST)-normalized expression, log2FC and adjusted p-values were calculated using DESeq2. (D) Pseudobulk module score of Hallmark TGF-β response gene set between WT and EC-KO grafts. Statistical significance was assessed using the Mann-Whitney U test. (E) Overlap of significantly differentially expressed genes in human (CAV+ vs. CAV-stable biopsy) and murine (WT vs. EC-KO) endothelial datasets. (F) Scatter plot of the 146 shared differentially expressed genes shared between the human and mouse EC datasets. Concordant genes are changing in the same direction in both datasets. Significance of concordance was tested using a binomial test (null hypothesis= 50% concordance), and Pearson correlation was used to assess correlation of gene expression changes across species. (G) Gene ontology (GO) enrichment analysis of concordant upregulated genes between human and mouse datasets. (H) GO enrichment analysis of concordant downregulated genes between human and mouse datasets.

bioRxiv preprint doi: https://doi.org/10.64898/2026.02.18.706637; this version posted February 19, 2026. The copyright holder for this preprint (which was not certified by peer review) is the author/funder, who has granted bioRxiv a license to display the preprint in perpetuity. It is made available under aCC-BY 4.0 International license.

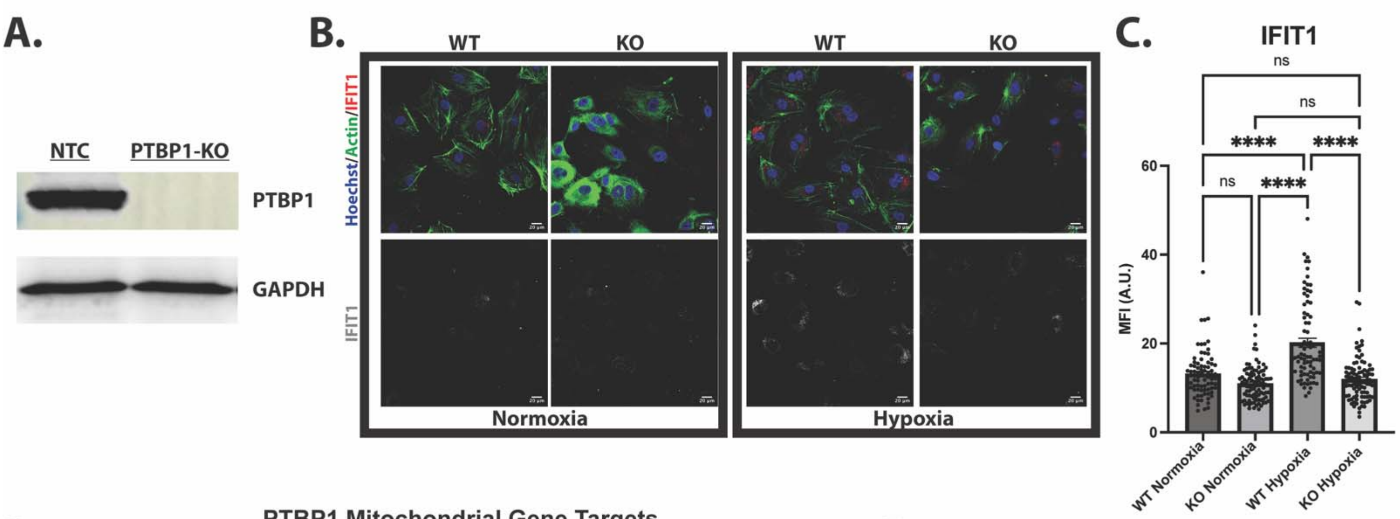

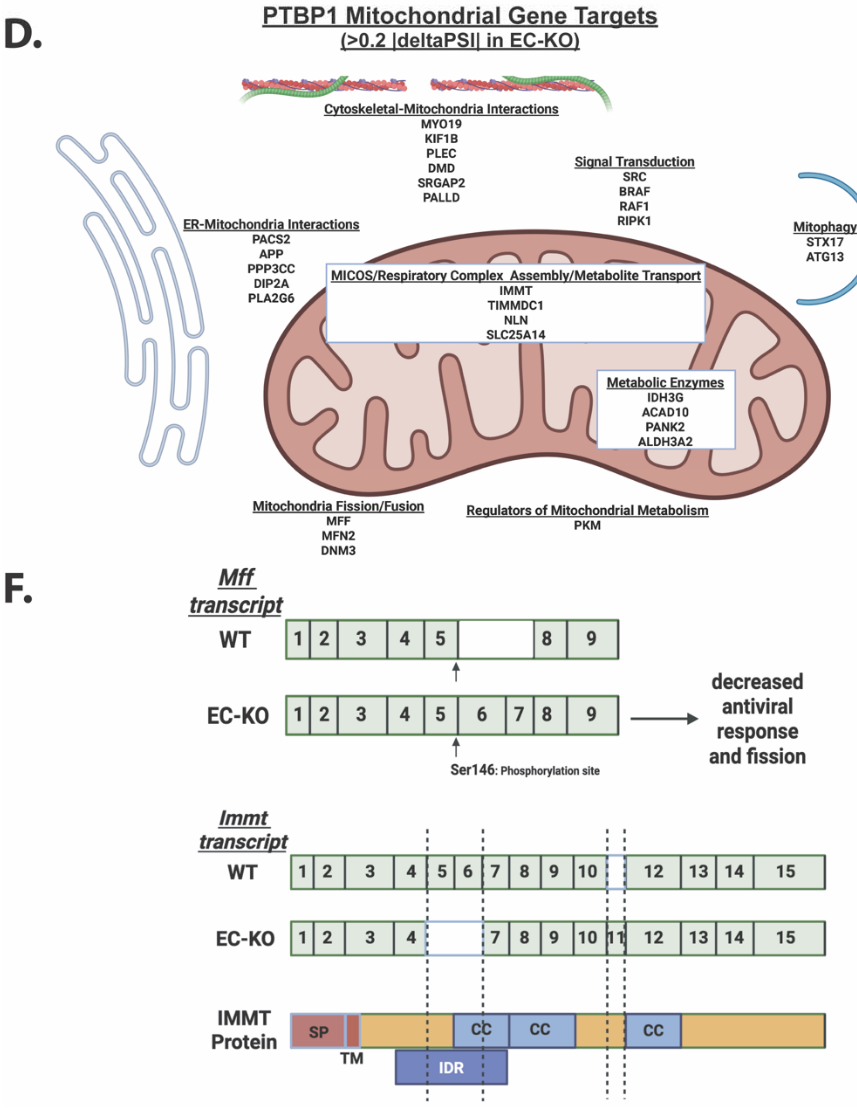

E.

Gene / Category	Max_abs_deltapsi
Regulators of Mito Metabolism	
PKM	0.610
Mito Fission/Fusion	
DNM3	0.257
MFF	0.289
MFN2	0.547
Cytoskeletal–Mito Interactions	
DMD	0.337
KIF1B	0.654
MYO19	0.229
PALLD	0.918
PLEC	0.786
SRGAP2	0.272
ER–Mito Interactions	
APP	0.359
DIP2A	0.367
PACS2	0.251
PLA2G6	0.229
PPP3CC	0.259
Signal Transduction	
BRAF	0.417
RAF1	0.245
RIPK1	0.817
SRC	0.332
Mitophagy	
ATG13	0.749
STX17	0.253
MICOS/Resp Complex/Transport	
IMMT	0.338
NLN	0.225
SLC25A14	0.239
TIMMDC1	0.448
Metabolic Enzymes	
ACAD10	0.298
ALDH3A2	0.361
IDH3G	0.401
PANK2	0.517
Other	
CCDC136	0.335
HIVEP1	0.356
KANSL3	0.915
OXR1	0.237

SI Figure 12. PTBP1 mediates splicing of endothelial mitochondrial transcripts. (A) Immunoblot of PTBP1 and GAPDH in non-targeting control (NTC) and PTBP1-knockout (KO) pooled HUVECs (B) Representative immunofluorescence images of WT and PTBP1 KO HUVECs cultured +/- hypoxia for 48 hrs and stained with IFIT1, phalloidin (actin), and Hoechst (upper panel). IFIT1 staining alone (grayscale) (bottom panel). (C) Quantification of IFIT1 fluorescence intensity. Each dot represents an individual cell from two biological replicates. Statistical significance was determined by one-way ANOVA with Tukey's multiple comparisons test. (D) Schematic overview of mitochondrial genes exhibiting significant alternative splicing (|deltaPSI| >0.2) in PTBP1 EC-KO murine endothelial cells. (E) Table of PTBP1-mediated spliced mitochondrial genes in isolated murine arterial endothelial cells shown as ΔPSI values. Data were reanalyzed from (Hensel *et al*., 2022). (F) Gene models showing alternative splicing changes in Mff and Immt gene upon EC-KO. Alignment of Uniprot protein domains with exons was performed using UCSC Genome Browser. SP: signal peptide, TM: transmembrane domain, IDR: intrinsically disordered domain, CC: coiled-coil domain. Schematics in (D) and (F) were made using BioRender.

bioRxiv preprint doi: https://doi.org/10.64898/2026.02.18.706637; this version posted February 19, 2026. The copyright holder for this preprint (which was not certified by peer review) is the author/funder, who has granted bioRxiv a license to display the preprint in perpetuity. It is made available under aCC-BY 4.0 International license.

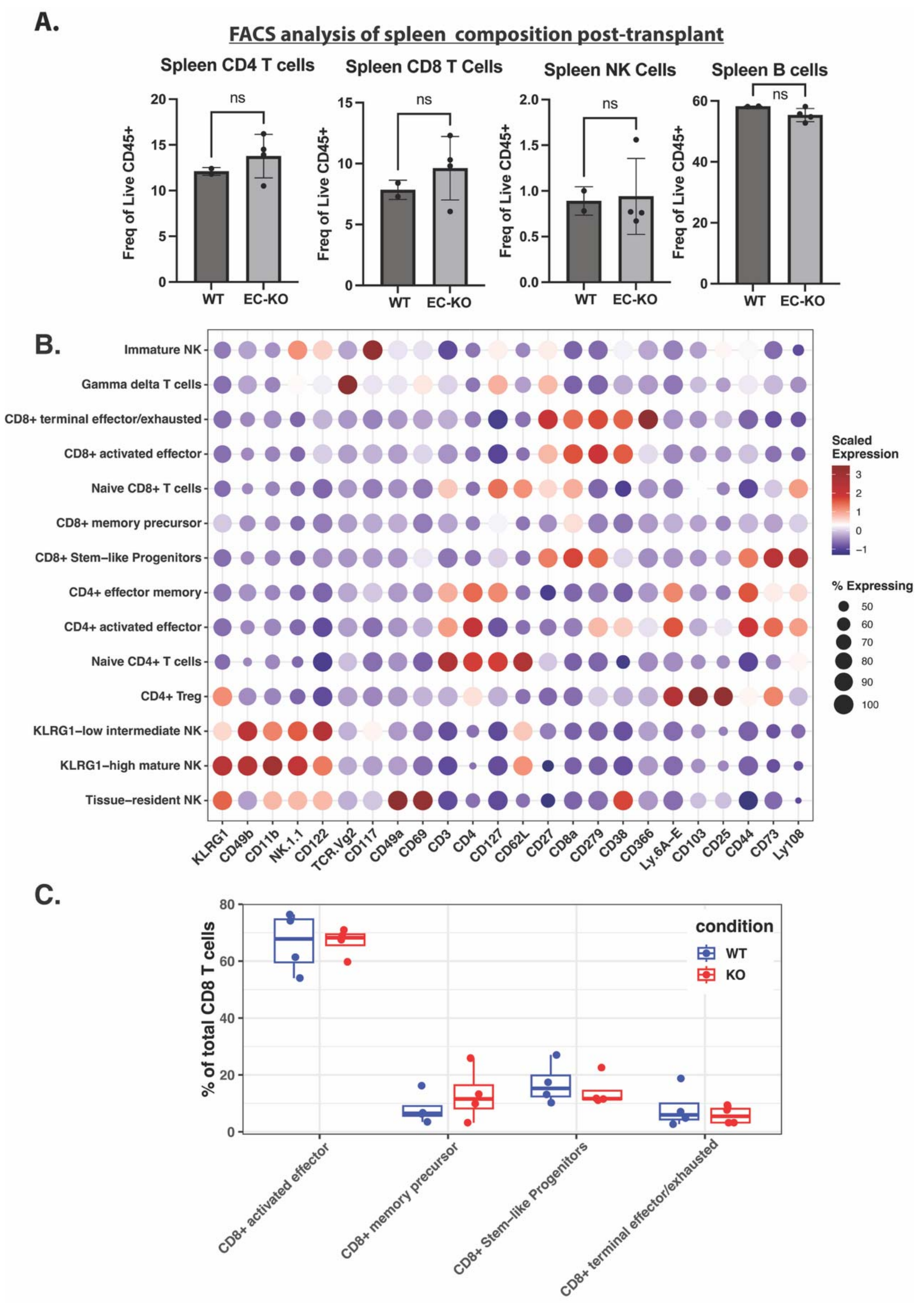

SI Figure 13. Spleen composition post-transplant and identification of lymphocyte subclusters in WT and EC-KO grafts using CITE-seq. (A) Flow cytometric quantification of CD4+ T cells, CD8+ T cells, NK1.1+ NK cells, and CD19+ B cells in spleens following transplantation (WT, n = 2; EC-KO, n = 4). Data are shown as mean ± SD; significance was assessed using an unpaired two-tailed Student's t test.(B) Dot plot of surface protein markers used to distinguish T and NK subtypes. Immature NK (NK.1.1+, CD122+, CD117+), Tissue-resident NK (NK1.1+, KLRG1+, CD49a+, CD69+, CD49b-), KLRG1-low intermediate NK (NK1.1+, KLRG1 low, CD11b+, CD49b+, CD122+), KLRG1-high mature NK (NK1.1+, KLRG1 high, CD11b+, CD49b+, CD62L high), Naïve CD4+ T cells (CD3+, CD4+, CD127+, CD62L+), CD4+ effector memory (CD3+, CD4+, CD44+), CD4+ activated effector (CD3+, CD4+, CD44+, Ly6A-E high, CD279+, CD38+, CD73+), CD4+ Treg (CD4+, CD103+, CD25+, KLRG1+) , Naïve CD8+ T cells (CD3+, CD8a+, CD127+, CD62L+), CD8+ memory precursor (CD8a+, CD127 low, CD62L-), CD8+ stem-like progenitor T cell (CD8a+, CD27+, CD279+, CD44+, CD73+,Ly108+), CD8+ activated effector T cells (CD8a+, CD27+ CD38+, CD279+), CD8+ terminal effector/exhausted T cells (CD8a+, CD27+ CD38+, CD279+, CD366+), Gamma-delta (TCR-VG+). (C). Proportion of CD8+ T cell subsets of total T cells per sample and condition (WT, n = 4, EC-KO, n =4).

bioRxiv preprint doi: https://doi.org/10.64898/2026.02.18.706637; this version posted February 19, 2026. The copyright holder for this preprint (which was not certified by peer review) is the author/funder, who has granted bioRxiv a license to display the preprint in perpetuity. It is made available under aCC-BY 4.0 International license.

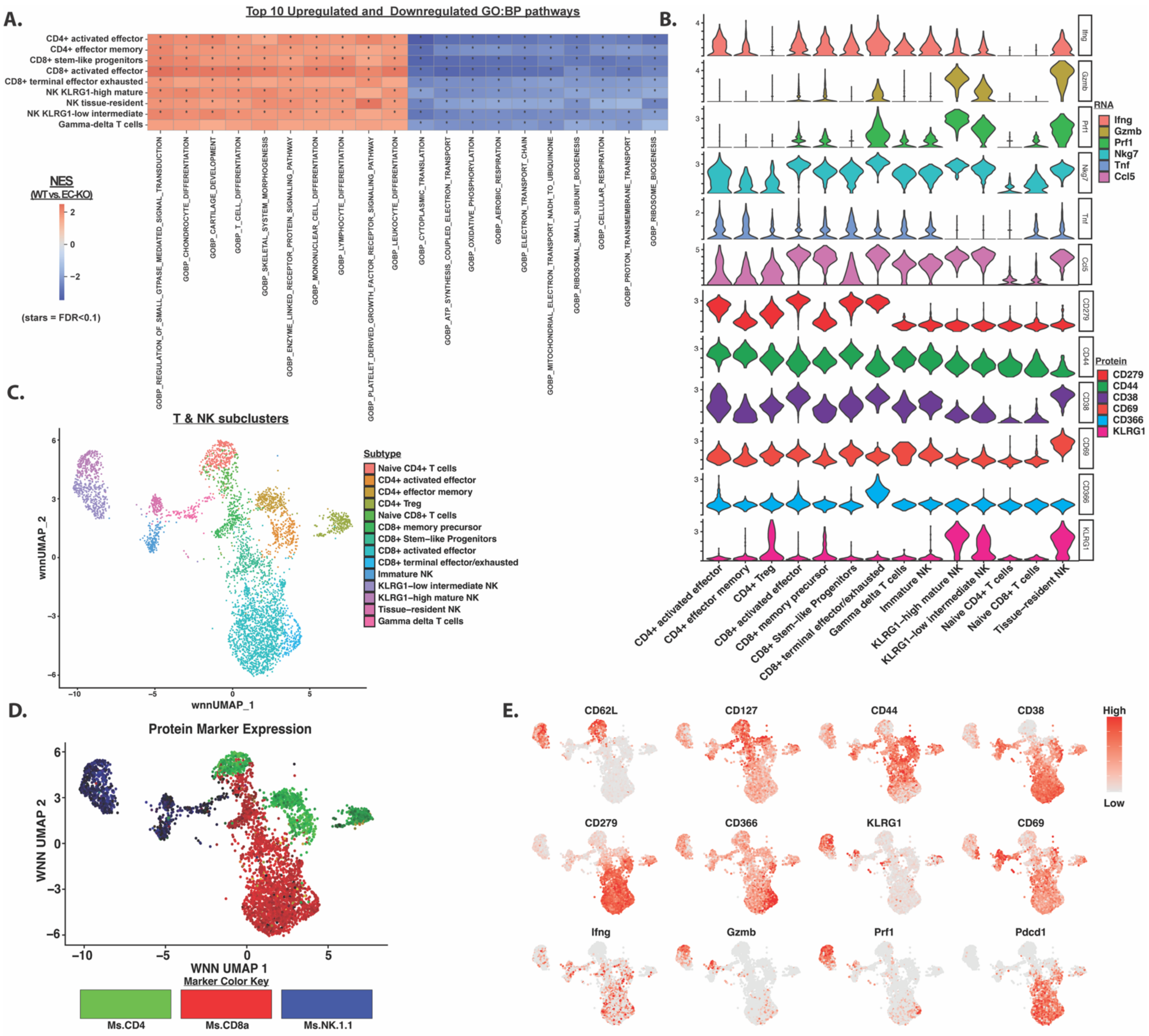

SI Figure 14. Differential immune signaling in T and NK subsets between WT and EC-KO grafts. (A) Heatmap showing the top 10 upregulated and downregulated gene ontology (GO) pathways in T and NK subsets in WT vs. EC-KO grafts. Normalized enrichment score (NES), stars denote pathways with FDR q value <0.1. (B) Violin plots of RNA and protein marker expression across T and NK cell subtypes. (C) UMAP of T and NK subtypes. (D) Feature plot of CD4, CD8a, and NK1.1 protein expression. (E) Feature plots of protein and RNA markers.

bioRxiv preprint doi: https://doi.org/10.64898/2026.02.18.706637; this version posted February 19, 2026. The copyright holder for this preprint (which was not certified by peer review) is the author/funder, who has granted bioRxiv a license to display the preprint in perpetuity. It is made available under aCC-BY 4.0 International license.

SI Tables:

For processing details see : https://github.com/pamurphyUCONN/2026_Pathoulas

SI Table 1. Donor Tissues.

BatchSouporcell – Batch and souporcell cluster (from pan-analysis of all samples)
Cellcount – cell count in cluster
UMI_count – unique transcripts counted (mean)
Gene_count – unique genes counted (mean)
Feature_count – unique antibodies counted (mean)
Endo_cellcount – endothelial cells
Endo_UMI_count – unique transcripts counted, in endothelial cells (mean)
Endo_Gene_count – unique genes counted, in endothelial cells (mean)
Endo_Feature_count – unique antibodies counted, in endothelial cells (mean)
souporcell_status – overall souporcell assignment
souporcell_assignment – pan-batch assignment
souporcell_statusBatch – most common 10x batch souporcell call (singlet or doublet)
souporcell_assignmentBatch - most common 10x batch souporcell call
souporcell_statusMerge – pan-batch status
10xRxn – 10x run batch
SampleType – biopsy or tissue explant
BankSource – bank of origin
Location – septal, ventricle
Donor – stripped ID (unassignable to PHI)
Day – Day after Tx (or 0 if no transplant)
ConfidenceOnHashing – confidence on hashing, high was only required in one sample for SNP assignment
DiseaseStatus – AMR, ACR, CAV etc
TimePoint – general timepoint
GraftHost – SNP determination of graft vs host
RA_mean – right atrial pressure
RV_systolic – right ventricle systolic pressure
RV_diastolic – right ventricle diastolic pressure
PA_systolic – pulmonary artery systolic pressure
PA_diastolic – pulmonary artery diastolic pressure
PA_mean – pulmonary artery mean pressure
PCW – pulmonary capillary wedge pressure
AV_O2 – arteriovenous oxygen difference
CO – cardiac output
CI – cardiac index
PVR – pulmonary vascular resistance
EF_preop – ejection fraction, pre-op
LocationFine – fine location (e.g. left and right septum)

SI Table 2. Pseudobulk DESeq2 analysis by major cell type and disease state, human heart tissues.

SI Table 3A-B. Pseudobulk GSEA analysis by major cell type and disease state, human heart tissues.

SI Table 4A-D. Pseudobulk DESeq2 analysis by nuclear PTBP1 levels, human heart tissues.

SI Table 5A-D. Pseudobulk GSEA analysis by nuclear PTBP1 levels, human heart tissues.

SI Table 6. CITE-seq surface protein markers

SI Table 7. Pseudobulk mRNA and surface protein DESeq2 analysis by major cell type comparing genotypes, murine heart tissues.

SI Table 8. Pseudobulk GSEA analysis by major cell type comparing genotypes, murine heart tissues.

SI Table 9. Concordant genes between human (CAV+ vs. CAV- stable biopsy) and murine (WT vs. EC-KO) endothelial datasets.

SI Table 10. GSEA analysis of concordant genes between human (CAV+ vs. CAV- stable biopsy) and murine (WT vs. EC-KO) endothelial datasets.

SI Table 11. Pseudobulk mRNA DESeq2 analysis in T and NK subtypes comparing genotypes, murine heart tissues.

SI Table 12. Pseudobulk GSEA analysis in T and NK subtypes comparing genotypes, murine heart tissues.

SI Table 13. Pseudobulk protein analysis in T and NK subtypes comparing genotypes, murine heart tissues.

SI Table 14. Key reagents.
